# Histological Findings in Infective Endocarditis—A Retrospective Cohort Study Conducted at “Dr. Carol Davila” Central Military Emergency University Hospital in Bucharest

**DOI:** 10.3390/life14121658

**Published:** 2024-12-13

**Authors:** Corina-Ioana Anton, Ion Ștefan, Sorin Duțulescu, Florica Stăniceanu, Cosmin Alexandru Buzilă, Alexia-Teodora Ștefan, Adrian Streinu-Cercel

**Affiliations:** 1 Department of Infectious Diseases, “Dr. Carol Davila” Central Military Emergency University Hospital, 134 Calea Plevnei, 010242 Bucharest, Romania; corina-ioana.anton@drd.umfcd.ro; 2 Department of Medico-Surgical and Prophylactic Disciplines, Titu Maiorescu University, 040441 Bucharest, Romania; 3 Faculty of General Medicine, Carol Davila University of Medicine and Pharmacy, 8 Eroii Sanitari Bvd, 050474 Bucharest, Romania; floriastaniceanu@yahoo.fr (F.S.); alexia-teodora.stefan0720@stud.umfcd.ro (A.-T.Ș.); adrian.streinucercel@umfcd.ro (A.S.-C.); 4 National Institute for Infectious Diseases “Prof. Dr. Matei Balş”, 1 Dr. Calistrat Grozovici Street, 021105 Bucharest, Romania; sorin.dutulescu@yahoo.com; 5 Cardiovascular Surgery Department, “Dr. Carol Davila” Central Military Emergency University Hospital, 134 Calea Plevnei, 010242 Bucharest, Romania; buzilacosmin@yahoo.com; 6 Department of Infectious Diseases I, Faculty of Medicine, Carol Davila University of Medicine and Pharmacy, 020021 Bucharest, Romania

**Keywords:** infective endocarditis, prosthetic valve endocarditis, pathology

## Abstract

Background: Histological findings of infective endocarditis (IEs) in mechanical valves present a complex diagnostic challenge owing to the lack of a precise definition. This ambiguity is further complicated by the natural degenerative processes that occur in the mechanical valves over time. Consequently, pathologists and clinicians face significant difficulties in distinguishing between genuine infective processes and the normal wear and tear of mechanical valves. Method: This retrospective cohort study was conducted between January 2017 and January 2024 and examined tissue samples from 93 patients who underwent a surgical removal of mechanical heart valves, with 41 cases suspected of infective endocarditis and 52 cases of non-IE. The researchers aimed to establish more precise histological criteria for distinguishing between these two conditions, focusing on two key features: vegetations and inflammatory patterns. Results: IE in patients with prosthetic heart valves presents distinct histological features that aid in the diagnosis and differentiation of non-infective complications. Hallmark characteristics include vegetation and inflammatory infiltrates with neutrophils. Valve tissue specimens from patients whose mechanical valves were removed because of non-infectious complications showed a different histological profile. Inflammatory infiltrates were observed in approximately 26% of these cases; however, they were primarily composed of macrophages and lymphocytes rather than neutrophils. Conclusions: By emphasizing neutrophil-rich inflammation as a key indicator, clinicians and pathologists could more effectively distinguish between true infective endocarditis and non-IE that can occur in the mechanical valves. This distinction is crucial for appropriate patient management as the treatment strategies for infective and non-infective valve conditions differ significantly.

## 1. Introduction

The incidence of infective endocarditis has been rising in recent years, largely due to the increasing use of vascular and cardiac devices, particularly in elderly individuals with comorbidities [1,2]. Left-sided valves are most commonly affected by vegetations, which often leads to valve failure. In contrast, right-sided valves were more commonly affected by intravenous drug use [2]. Over the years, the causative organisms have shifted, with a growing incidence of *Staphylococcus* species [3].

IE is a complex medical condition that can present with a variety of symptoms including fever, heart failure, and stroke. Clinical suspicion is essential for proper diagnosis and treatment in individuals with risk factors. This condition can also lead to severe complications such as myocardial infarction. The accurate diagnosis of IE requires a comprehensive approach that integrates clinical, imaging, and laboratory analyses [3,4].

Laboratory diagnostic techniques for infective endocarditis encompass a range of methods, each of which has its own strengths and applications. Serological tests are used to detect antibodies produced in response to specific pathogens, thereby providing indirect evidence of infection [2,3]. These tests are particularly useful for identifying certain organisms that are difficult to culture, such as *Coxiella burnetii* or *Bartonella species*. Molecular methods, including polymerase chain reaction (PCR) and gene sequencing, offer high sensitivity and specificity for detecting microbial DNA and RNA [4]. These techniques are especially valuable when blood cultures are negative or patients have received prior antibiotic treatment [2,3,4].

The pathological examination of valve tissue samples remains the gold standard for diagnosing infective endocarditis when such specimens are available. This method allows the direct visualization of microorganisms and associated inflammatory changes within the affected tissue. Histopathological analysis can reveal characteristic features such as vegetation formation, inflammatory cell infiltration, and tissue destruction [4].

The criteria for this diagnosis encompass histological demonstration of pathological lesions, such as vegetation and inflammatory infiltrates [5].

Pathological examination of prosthetic heart valves is considered to be one of the most accurate methods for diagnosing IE. However, the identification of causative agents is challenging [4,5].

PVE is a severe condition that poses a significant threat to both individual and societal well-being as it is associated with extended hospitalization periods, substantial socioeconomic factors, and diminished quality of life [5].

Recently, there has been a notable increase in the incidence of infective endocarditis (IE) in adults. Several factors have been proposed to contribute to this increase, including an aging population, the growing prevalence of cardiac surgery for prosthetic heart valves, and increase in the use of cardiac electronic devices [5,6].

Although some of these factors have been found to be unrelated to the increased incidence, there is a need for a comprehensive evaluation of this disease in patients with prosthetic heart valves [6,7].

The annual incidence of PVE around the globe is approximately 2%, and it is generally regarded as an uncommon complication that may occur following valve replacement surgery [6,7].

Individuals who have undergone valve replacement surgery or transcatheter valve replacement are at a higher risk of developing PVE because of the presence of a prosthetic valve, which can become infected and cause severe complications if not treated promptly. Therefore, it is crucial for healthcare providers to consider PVE a potential diagnostic tool when assessing patients with IE [7,8].

PVE is a serious and potentially life-threatening condition that has historically posed significant challenges to resource utilization and treatment improvement. The clinical outcomes associated with PVE have evolved with changes in underlying causes [8,9].

In recent years, the surgical repair of cardiac valves has emerged as the preferred treatment option for many patients with heart valve dysfunction [8,9,10].

This shift in approach is primarily due to significant improvements in patient outcomes, including higher survival rates and enhanced quality of life post-surgery. Advancements in surgical techniques coupled with a better understanding of valve anatomy and function have contributed to the increased success of these procedures [9,10]. Patients undergoing valve repair often experience fewer complications, reduced need for long-term anticoagulation therapy, and improved preservation of heart function compared to those receiving valve replacement [9,10].

The diagnosis and treatment of prosthetic valve endocarditis (PVE) relies heavily on the Duke criteria, which emphasizes the importance of echocardiography and blood cultures [10]. Transesophageal echocardiography is particularly valuable for detecting vegetation and offers high sensitivity and specificity [5,6,7].

However, the absence of vegetation does not necessarily rule out infective endocarditis; conversely, degenerative changes in mechanical valves can be mistaken for vegetation. Blood cultures, which are crucial for identifying the causative organism, may yield false-negative results if the patient has recently received antibiotics or if the infecting organism is fastidious or requires special culture techniques [9,10].

Alternative methods have been developed to address these challenges. Serological tests can be used to detect specific antibodies or antigens that are associated with certain pathogens [9].

Pathological examination remains the gold standard for confirming IE when such specimens are available. Histological findings from these examinations are incorporated into the Duke criteria and considered definitive evidence of infective endocarditis [10,11]. These complementary approaches help to improve diagnostic accuracy in cases where traditional methods may be inconclusive, ensuring that patients with suspected PVE receive appropriate and timely treatment [11].

This study aimed to establish more precise histological criteria for distinguishing between these two conditions, focusing on two key features: vegetation and inflammatory patterns.

## 2. Materials and Methods

### 2.1. Study Design

This study was a population-based cohort analysis that utilized data from individuals who were admitted to “Dr. Carol Davila” Central Military Emergency University Hospital in Bucharest between 1 January 2017 and 1 January 2024. The investigation followed ethical guidelines and was authorized by the Ethics Committee of the “Dr. Carol Davila” Central Military Emergency University Hospital in Bucharest (Decision No. 562/20.12.2022). Informed consent was obtained from all the patients included in this study.

### 2.2. Setting

Paraffin-embedded tissue sections were cut at 4–5 µm thickness and placed in a 60 °C oven for 1–2 h. Deparaffinization and rehydration were performed using graded alcohol concentrations (100%, 95%, and 70%), followed by distilled water. For CD68 staining, heat-mediated antigen retrieval using EDTA (pH 8.0) was performed using a Leica BOND III platform for 20 min. Antigen retrieval was not required for MPO staining. The RTU CD68 antibody (clone 514H12) or the MPO RTU antibody (clone 59A5) was applied for 30–60 min at room temperature and then washed with BOND Wash Solution (3 washes for 3–5 min each). The BOND Polymer Refine Detection Kit was used, followed by DAB substrate and closely monitored for optimal staining. Counterstaining was performed with hematoxylin for 1–3 min, followed by rinsing with water. The sections were dehydrated using graded alcohols, cleared in xylene, and mounted in a suitable medium. Microscopic evaluation was conducted to assess the quality and specificity of staining. The CD68 antibody specifically labels macrophages and monocytes, whereas the MPO antibody targets myeloperoxidase, an enzyme primarily found in neutrophils and monocytes. The evaluation process likely involved examining the tissue sections under various magnifications to observe the distribution and intensity of DAB (brown) staining, which indicated the presence of the target antigens. Counterstaining with hematoxylin provided a blue background, allowing for a better visualization of the tissue architecture and cellular morphology. This evaluation would help to determine the effectiveness of the staining protocol and provide insights into the presence and distribution of macrophages, monocytes, and neutrophils within tissue samples. The immunohistochemistry protocol is a standard method for detecting specific proteins in tissue sections. This involves a series of carefully controlled steps to ensure optimal staining results. The automated staining system of the Leica BOND III platform and BOND Polymer Refine Detection Kit can enhance reproducibility and consistency across multiple samples. The specific antibodies used (CD68 and MPO) are well-established markers for identifying inflammatory cells, particularly those involved in the innate immune response. This staining technique allows researchers to visualize and quantify these cell types within the context of the tissue microenvironment, providing valuable information about inflammatory processes, immune cell infiltration, and potential disease mechanisms.

The specified approach involved obtaining five distinct sections from each patient for histological examination, with two images captured per section. This systematic sampling method allows for comprehensive analysis of tissue structure and composition across multiple areas of interest within a single patient.

### 2.3. Study Population

Between January 2017 and January 2024, a comprehensive study was conducted at “Dr. Carol Davila” Central Military Emergency University Hospital in Bucharest, focusing on patients who underwent surgical removal of mechanical heart valves. The study involved 93 patients who were divided into two distinct groups based on their preoperative diagnoses. The first group, comprising 41 patients, was classified into the endocarditis group. These patients were diagnosed with either definite or possible infective endocarditis according to the Duke criteria, which were applied preoperatively without considering the pathological criteria. The second group, termed the control group, comprised 52 patients who underwent valve removal due to presumed non-infective dysfunction. Importantly, these patients tested negative for infective endocarditis based on preoperative application of the Duke criteria. The study methodology was rigorous, with confirmed cases of infective endocarditis determined by the detection of microorganisms through standard blood culture samples or valve material analysis. The research team, consisting of physicians and surgeons at the hospital, collected and analyzed a wide range of data. This included detailed information on the patients’ clinical status, echocardiographic findings, preoperative diagnoses based on the Duke criteria, and valve tissue samples. This comprehensive approach allowed for a thorough examination of the differences between the infective and non-infective causes of mechanical heart valve removal, potentially providing valuable insights into the diagnosis and management of these complex cardiac conditions.

### 2.4. Statistical Analysis

The Mann–Whitney U test was applied to assess the statistical significance of the results for each histological parameter of the valve tissue samples from patients in the early onset and late-onset groups. The Kruskal–Wallis test yielded *p*-values, which indicate the probability of obtaining differences as significant or greater than those observed in our data, assuming that the null hypothesis is valid. If the *p*-value was less than the predetermined significance level of 0.05, we rejected the null hypothesis and concluded that there were significant differences between at least two groups. Statistical analyses were performed using the SPSS software version 26.

## 3. Results

The study compared two groups: an infective endocarditis group consisting of forty-one prosthetic valve specimens and a group with fifty-two specimens of non-infective dysfunction prosthetic heart valve. The demographic analysis revealed notable differences between the two groups. Patients in the endocarditis group were generally younger, with a mean age of 62.40 years (SD ± 18.60), compared to the control group, which had a mean age of 74.10 years (SD ± 21.33).

Both groups showed a higher proportion of male patients, with the endocarditis group having a male-to-female ratio of 1.34 (29 men and 11 women) and the control group having a ratio of 1.71 (41 men and 11 women). The distribution of valve involvement also differed significantly between the two groups. In the IE group, aortic valves were more frequently affected, accounting for 67.1% of the cases, while mitral valves were involved in 32.9% of the cases. Conversely, in the non-infective dysfunction prosthetic heart valve group, mitral valve involvement was more common, occurring in 58.2% of patients, whereas aortic valve involvement was observed in 41.8% of cases. These findings suggest that infective endocarditis may have a predilection for aortic valves, whereas non-infective endocarditis appears to affect the mitral valves more frequently. This difference in valve involvement patterns could have implications in the diagnosis, treatment, and prognosis of prosthetic valve endocarditis [12].

Mechanical valve endocarditis is primarily caused by bacterial and fungal pathogens, with Staphylococci and Streptococci being the predominant causative agents [12,13]. In this study, *Staphylococcus aureus* emerged as the most common pathogen responsible for ten cases. This was followed by *Enterococcus faecalis*, which caused six cases. *Staphylococcus* epidermidis accounted for three cases, whereas *Streptococcus mitis* was responsible for two additional cases. These findings underscore the significance of Gram-positive bacteria in metallic valve endocarditis. Interestingly, fungal infections, although less common, were observed. One case of metallic valve endocarditis was attributed to Candida albicans, highlighting the potential of fungal pathogens to cause this condition. 

This diverse range of causative organisms emphasizes the importance of accurate microbiological identification for the diagnosis and treatment of IE. The prevalence of staphylococcal and streptococcal infections suggests that targeted antimicrobial therapies against these pathogens may be crucial for the effective management of this condition.

### 3.1. Histological Findings

**Figure 1:**
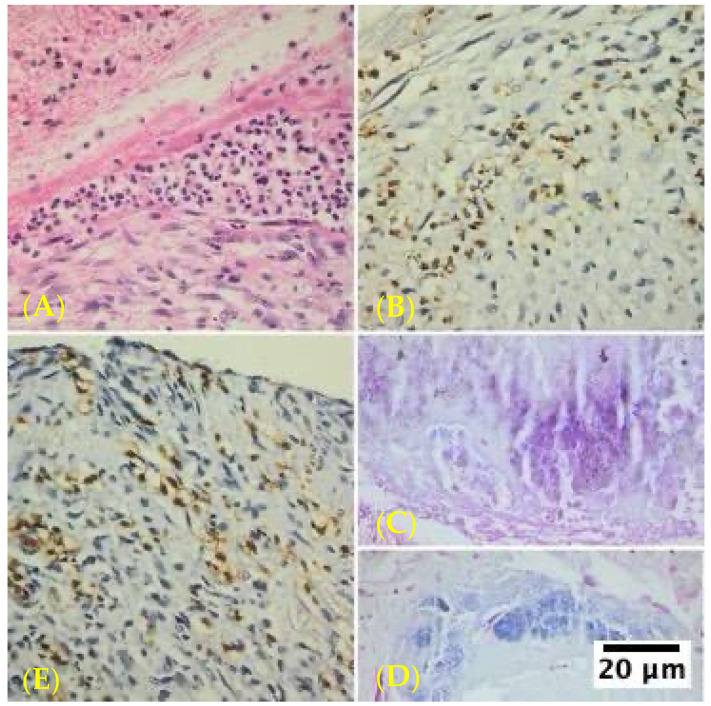
Infective endocarditis in mechanical heart valve tissue specimens in aortic position using different staining examined under high magnification (400×) using Olympus BX43 microscope and XC30 camera. The predominance of neutrophils in the inflammatory infiltrates provides valuable insights into the acute nature of the infection and the body’s immediate immune response. (**A**) Hematoxylin–eosin staining, inflammatory infiltrates with numerous neutrophils and tissue fibrosis. (**B**) MPO-positive neutrophil cells; numerous neutrophils are present in both the superficial fibrin network and subendocardial tissue of a mitral position mechanical heart valve. (**C**) Gram stain of *Staphylococcus aureus* PVE demonstrating Gram-positive cocci. Inconsistent staining patterns are frequently observed in tissue sections that have been Gram-stained. Gram 400× deparaffinization artifacts. Gram-positive microbial colonies; subendothelial conjunctive tissue; surface fibrin deposits; black–brownish deposit artifacts (**D**) Gram stain demonstrating *Stahpylococcus aureus* PVE; fine-grained blue structures arranged in clusters; (**E**) numerous MPO-stain-positive neutrophils (brown precipitate) in the subendocardial tissue of an aortic position mechanical heart valve.

The study revealed distinct histopathological characteristics between IE and non-infective valve dysfunction. The majority of IE cases (87.8%) showed significant inflammatory infiltrates, primarily composed of neutrophils, indicating an active infection. The infective process was predominantly localized to the vegetation on the valve cusp surface, with minimal involvement of the underlying valve tissues. Microorganisms were visually identified in approximately half of the infective endocarditis samples using special staining techniques (Figure 1).

**Figure 2:**
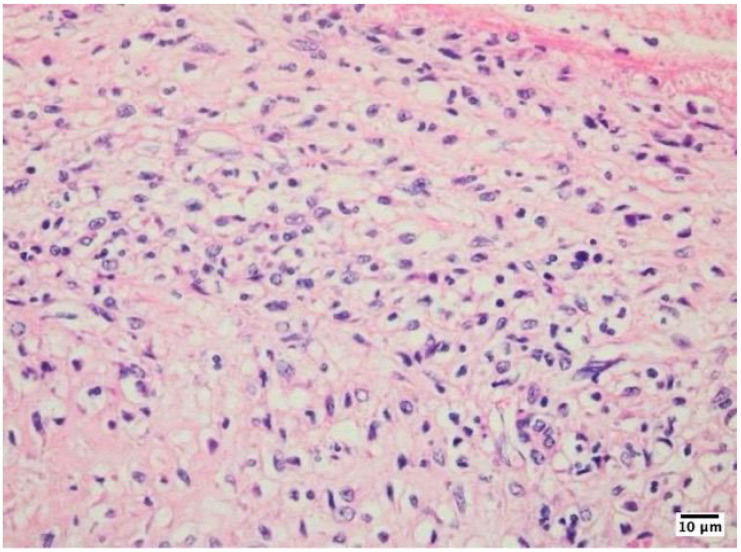
Histological examination of a mechanical heart valve tissue specimen in mitral position showed non-infective inflammatory degenerative lesions as visualized using hematoxylin–eosin staining at 400× magnification with an Olympus BX43 microscope and XC30 camera. This finding is characterized by the presence of numerous macrophages lining the area surrounding the mechanical valve. The accumulation of these immune cells suggests an ongoing inflammatory process, which may be a response to the presence of the artificial valve or a result of chronic tissue irritation.

**Figure 3:**
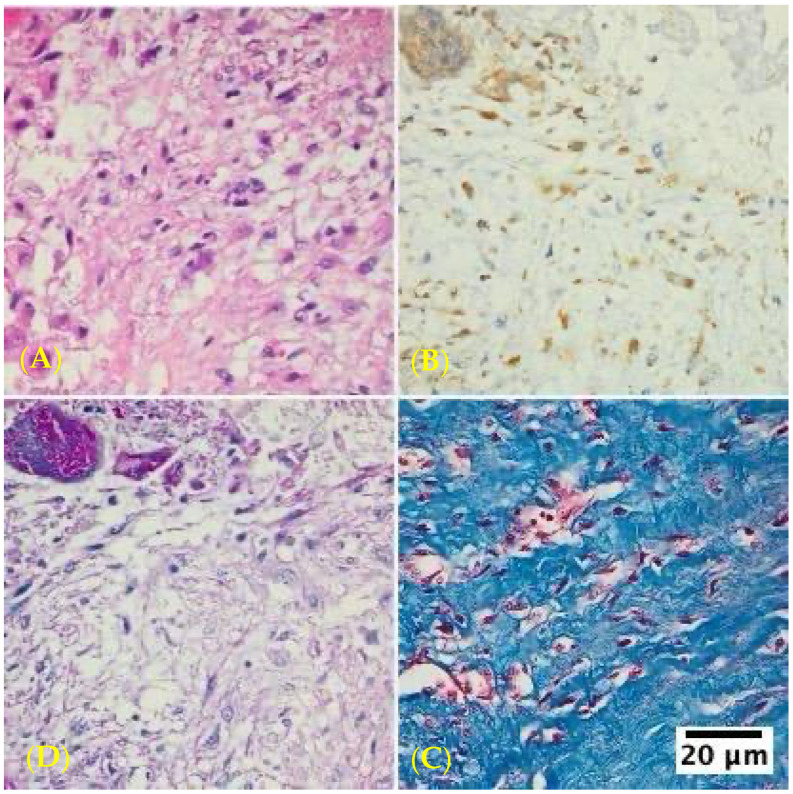
Histological examination of an aortic position mechanical valve tissue specimen with non-infective inflammatory degenerative lesions as visualized using different staining at 400× magnification with an Olympus BX43 microscope and XC30 camera. (**A**) Hematoxylin–eosin staining, inflammatory infiltrates with numerous macrophages. (**B**) CD68 staining showing inflammatory infiltrates with macrophages. (**C**) Masson Trichrome staining, marked interstitial fibrosis. (**D**) PAS staining showing inflammatory infiltrates with macrophages.

**Figure 4:**
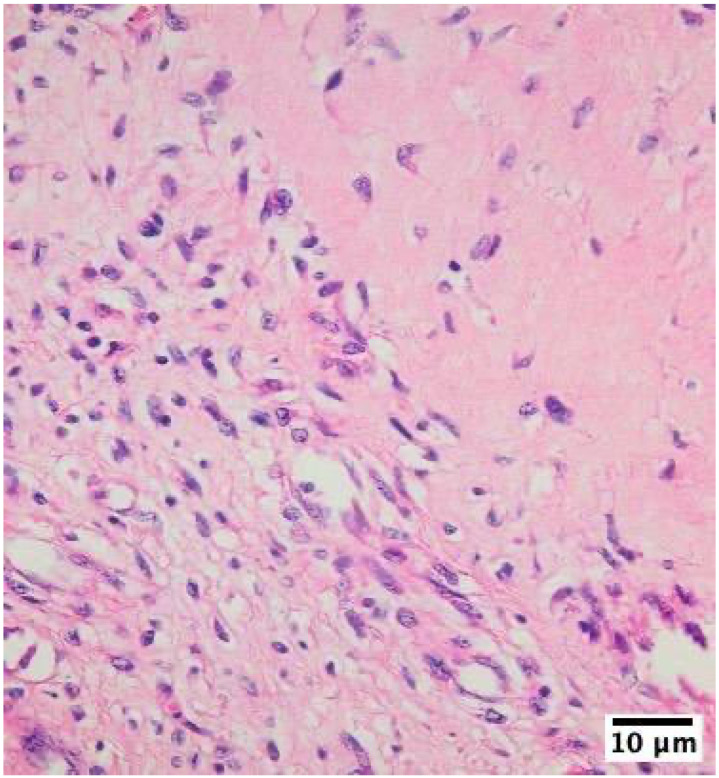
Histological examination of mechanical heart valve tissue specimen in mitral position with non-infective inflammatory degenerative lesions showing inflammatory infiltrates with lymphocyte and macrophage cells as visualized using hematoxylin–eosin–saffron staining at 400× magnification with an Olympus BX43 microscope and XC30 camera.

The inflammatory infiltrates in non-IE cases consisted mainly of macrophages and lymphocytes, suggesting a chronic inflammatory process rather than an acute infection (Figure 2, Figure 3 and Figure 4).

This finding highlights the potential for misdiagnosis in both histological examinations and echocardiography, emphasizing the importance of careful differentiation between true infective vegetation and non-infective thrombi to ensure accurate diagnosis and appropriate treatment strategies.

In contrast, vegetation was exclusively detected in the IE group, serving as a key differentiating factor between these two conditions. Vegetations occupied a substantial portion of the valve tissue area, accounting for an average of 61.9%. This significant presence of vegetation in infective cases underscores its importance as a diagnostic marker for infective endocarditis in mechanical valves.

The absence of vegetation in non-infective cases further emphasizes their specificity as an indicator of infection, potentially aiding in more accurate and timely diagnosis of infective endocarditis in patients with mechanical heart valves [13].

The distribution of pathogens in patients with confirmed IE provides valuable insights into the microbial etiology of these patients. In this study, *S. aureus* emerged as the predominant causative agent, accounting for 34.1% of cases (14 patients). This finding aligns with the global trend that *S. aureus* is a leading cause of IE, likely due to its virulence factors and the increasing prevalence of healthcare-associated infections. *E. faecalis* was the second most common pathogen, identified in 19.5% of cases (eight patients), highlighting the growing importance of enterococci in IE. *S. mitis* was found in 7.3% of cases (three patients), representing a smaller but significant proportion of IE cases. These findings underscore the shifting landscape of IE etiology, with *S. aureus* surpassing streptococci as the primary causative agent in many regions. The relatively high prevalence of *E. faecalis* is noteworthy and may reflect the increasing incidence of enterococcal infections, particularly in healthcare settings or among individuals with specific risk factors. Although less frequent, the presence of *S. mitis* serves as a reminder of the continued relevance of oral streptococci in IE pathogenesis. The distribution of pathogens has important implications for empirical antibiotic therapy, diagnostic approaches, and preventive strategies in the management of IE.

### 3.2. Quantified Data for Neutrophils, Lymphocytes, and Macrophages

The analysis of the neutrophil counts revealed striking differences. In IE patients, the mean neutrophil count per high-power field (HPF) was substantially higher, at 120 ± 25 cells, with a range of 90–160 cells/HPF. These neutrophils constituted a significant majority of the total cellular infiltrates, accounting for 75 ± 5%. This high neutrophil count suggests an intense inflammatory response characteristic of IE. In contrast, patients without IE exhibited markedly lower neutrophil counts. The mean cell count per HPF was 25 ± 10 cells, ranging from 10 to 40 cells/HPF. Neutrophils in these patients accounted for only 20 ± 4% of the total infiltrates. The stark difference between the two groups was further emphasized by statistical analysis, which yielded a *p*-value of <0.001. This highly significant result indicates that the increased neutrophil presence in IE patients is not due to chance but is a reliable marker of the disease state. These findings underscore the importance of neutrophil-mediated inflammation in the pathophysiology of IE and suggest that the neutrophil count could be a valuable diagnostic indicator for distinguishing IE from other conditions 2.

The analysis of lymphocyte infiltration revealed significant differences in both cell counts and proportions. In IE patients, the mean lymphocyte count per HPF was substantially higher, at 90 ± 20 cells, with a range of 70–110 cells/HPF. These lymphocytes constituted a large majority of the total cellular infiltrates, accounting for 82% ± 4%. This indicates a pronounced lymphocytic response in the affected cardiac tissues of patients with IE. In contrast, non-IE patients exhibited markedly lower lymphocyte counts, with a mean of 15 ± 12 cells per HPF and a range of 15–45 cells/HPF. The proportion of lymphocytes among the total infiltrates was also considerably lower in non-IE patients (30% ± 5%). The stark difference between these two groups is further emphasized by the highly significant *p*-value (*p* < 0.001), which strongly suggests that increased lymphocyte presence is a characteristic feature of inflammatory endocarditis. These data underscore the importance of lymphocytic infiltration in the pathophysiology of IE and may have implications for diagnosis and treatment strategies.

The analysis of macrophage presence revealed significant differences between the two groups. In IE patients, the mean macrophage cell count per HPF was 20 ± 8 cells, with a range of 10–35 cells/HPF. These macrophages constituted approximately 10% ± 3% of the total cellular infiltrates. In contrast, non-IE patients exhibited a markedly higher macrophage presence, with a mean cell count of 40 ± 12 cells per HPF and a range of 25–55 cells/HPF. Macrophages in non-IE patients represented a substantially larger proportion of the total infiltrates, accounting for 40% ± 6%. The statistical analysis of these findings yielded a *p*-value of less than 0.05, indicating a statistically significant decrease in macrophage presence in IE patients compared to that in non-IE patients. This substantial difference in macrophage populations between the two groups suggests that IE may be associated with altered immune responses or tissue-remodeling processes. The reduced presence of macrophages in IE patients could potentially impact various aspects of the disease, including inflammation regulation, tissue repair, and pathogen clearance. These findings may have important implications for understanding the pathophysiology of IE and could potentially inform future diagnostic and therapeutic approaches.

IE patients show a significantly higher number of neutrophils in their inflammatory infiltrates compared to non-IE patients, consistent with active infection. A significant difference in lymphocyte counts between the two groups, suggesting that lymphocytic infiltrates are a distinguishing feature of non-IE inflammation. Non-IE patients have significantly more macrophages than IE patients, which could reflect differences in the inflammatory response, such as chronic inflammation or healing in non-IE conditions. A higher percentage of neutrophils in IE patients reflects an acute bacterial infection, whereas higher macrophage counts in non-IE patients might indicate a more chronic inflammatory or healing process.

## 4. Discussion

Mechanical heart valves, while being lifesaving for many patients, can experience a range of durability issues and complications. These problems include material degeneration, calcification, perforation, paravalvular leak, partial dehiscence, thrombus formation, and infection [14]. Among these, infection remains a particularly serious complication with a high mortality rate despite advancements in medical and surgical treatments [13,14].

To better understand and standardize the histological features of infective endocarditis, a comprehensive analysis was conducted on a large number of mechanical valves removed because of IE or non-infective degenerative changes. The study revealed that, after implantation, heart valves develop a covering layer composed of fibrin, platelets, and erythrocytes.

Importantly, inflammatory infiltrates were observed in approximately 81.8% of non-infective prosthetic valves removed because of dysfunction, highlighting the need to differentiate between infection and normal inflammatory responses. The presence of acute inflammation, characterized by neutrophils in the valvular inflammatory infiltrates, strongly suggests infection.

In contrast, chronic inflammation, marked by infiltrates primarily composed of macrophages and lymphocytes, was observed in some samples. These findings contribute to a more nuanced understanding of the histological features associated with IE in mechanical heart valves, potentially improving the diagnostic accuracy and treatment strategies.

The inflammatory infiltrates observed in the cardiac valves during various pathological processes provide crucial insights into the underlying disease mechanisms. Neutrophils, macrophages, and T lymphocytes are commonly found in these infiltrates, and their relative proportions can provide valuable diagnostic information [11]. Notably, the presence of neutrophils in significant numbers appears to be a key differentiating factor for IE [11,15]. In contrast, non-infective valve processes, although potentially associated with substantial inflammatory infiltrates, typically exhibit different cellular compositions [15]. These infiltrates are predominantly composed of mononuclear leukocytes, macrophages, and T lymphocytes, with only a small proportion of neutrophils [11,15].

This distinction in cellular composition enables pathologists to make informed judgments about the likelihood of infective endocarditis using standard histological techniques [15,16]. When a predominance of neutrophils is observed in valvular inflammatory infiltrates during histological examination, it strongly suggests the presence of infective endocarditis [16]. This finding underscores the importance of careful histological analysis in guiding the accurate diagnosis and appropriate treatment strategies for patients with cardiac valve pathologies.

The presence of vegetation and microorganisms in valve tissue is a crucial criterion for histological diagnosis. Our study revealed that, when present, vegetation can occupy a significant portion of the valve tissue area, accounting for 61.9% of our findings. This substantial coverage underscores the potential severity of the infection and its impact on valve structure and function [17]. However, it is important to note that vegetation may be absent or small in some cases, covering only a minimal portion of the valve [17,18].

This variability in vegetation size and presence highlights the complexity of infective endocarditis and the need for careful examination of valve tissue samples. The size and extent of vegetation can provide valuable information regarding the progression and severity of infection [18]. Larger vegetation may indicate a more advanced or aggressive form of endocarditis, potentially increasing the risk of complications, such as embolization or valve destruction [16,17]. Conversely, smaller vegetation or their absence does not necessarily rule out IE, emphasizing the importance of considering other diagnostic criteria and clinical findings [17].

Additionally, preoperative antibiotic treatment can potentially destroy microorganisms, making them undetectable by histological methods. This presents a significant challenge in the diagnosis and management of IE because the absence of visible microorganisms in histological samples may lead to false-negative results [19]. The impact of antibiotic therapy on the detectability of microorganisms underscores the importance of timing sample collection and the need for a comprehensive diagnostic approach that integrates clinical, microbiological, and histological data. In our study, all patients diagnosed with infective endocarditis exhibited vegetation [19].

This finding suggests that larger vegetation may be associated with a higher likelihood of definitive diagnosis. However, it is crucial to consider that vegetation size alone should not be the sole criterion for diagnosis, as smaller vegetation or even its absence can occur in some cases of infective endocarditis.

At “Dr. Carol Davila” Central Military Emergency University Hospital in Bucharest, the handling of excised valve tissue samples follows a specific protocol designed to maximize diagnostic yield across multiple disciplines. Initially, the bacteriologist processes the samples, dividing them into selected portions for various bacteriological procedures, including culture systems.

This approach allows for comprehensive microbiological assessment, which is crucial for identifying the causative organism and guiding appropriate antibiotic therapy. Consequently, the remaining valve tissue samples submitted for histological examination are often small. This can result in challenges during histological analysis, as the reduced sample size may only show nonspecific inflammation, potentially limiting diagnostic accuracy.

The limited tissue available for histological examination may have led to sampling bias, as the most affected areas of the valve may not be represented in the examined sections. This underscores the importance of careful sample selection and processing to maximize the diagnostic yield of available tissue. The combination of preoperative antibiotic treatment and the division of samples for multiple tests highlights the importance of a comprehensive approach to diagnose IE, integrating clinical, microbiological, and histological findings [19].

This multidisciplinary approach is essential for overcoming the limitations of individual diagnostic methods and ensuring the accurate diagnosis and appropriate management of IE. To enhance diagnostic accuracy in cases with limited tissue samples or when preoperative antibiotic treatment may have affected the detectability of microorganisms, additional techniques such as immunohistochemistry or molecular methods such as polymerase chain reaction (PCR) may be employed [19,20].

These advanced techniques can potentially detect microbial antigens or genetic material, even in cases where traditional histological methods fail to identify microorganisms [20]. Furthermore, the correlation of histological findings with clinical presentation, echocardiographic data, and blood culture results is crucial for the comprehensive evaluation of suspected infective endocarditis cases [20].

This integrated approach can help overcome the limitations of individual diagnostic modalities and provide more accurate and timely diagnosis, ultimately improving patient outcomes through appropriate and targeted treatment strategies [19,20]. The presence of vegetation and visible microorganisms in valve tissue is a crucial criterion for histological diagnosis of IE [21].

Adherent thrombi on prosthetic heart valves are a potentially misleading phenomenon in cardiovascular medicine. These non-infectious, degenerative structures can form on the surface of artificial valves, mimicking the appearance of infectious vegetation when observed through echocardiographic imaging [19]. This similarity in appearance can lead to diagnostic challenges, potentially resulting in misdiagnosis and inappropriate treatment strategies. The formation of these thrombi is believed to be a result of altered blood flow dynamics around the prosthetic valve coupled with the inherent thrombogenicity of the artificial materials used in valve construction [20].

The distinguishing feature of adherent thrombi lies in their inflammatory profiles. Unlike infectious vegetation, which typically contains a diverse array of inflammatory cells, the inflammatory infiltrates associated with these thrombi are predominantly composed of macrophages [21,22].

This macrophage-rich environment suggests a chronic inflammatory response to the presence of prosthetic material or altered hemodynamics rather than an acute infectious process [23]. Understanding this distinction is crucial for clinicians because it affects both the diagnostic approach and management strategy.

Although infectious vegetation often requires aggressive antibiotic therapy and potential surgical intervention, adherent thrombi may be managed differently, potentially with anticoagulation therapy or close monitoring, depending on their size and clinical impact [24].

## 5. Conclusions

Histological analysis of mechanical valve tissues provides valuable insights into IE characteristics. This study addresses the challenges in diagnostics, particularly in cases where traditional indicators such as vegetation or visible microorganisms are absent. This research emphasizes the importance of inflammatory infiltrates, specifically those predominantly composed of neutrophils, as key markers for infective processes in prosthetic valves.

This finding is significant because it offers a more nuanced approach to distinguish between infective and non-infective inflammatory conditions in valve tissues. The implications of this study extend beyond mere observation, suggesting a potential need to revise the pathological Duke criteria for IE diagnosis.

By highlighting the role of neutrophil-dominated inflammatory infiltrates, this study proposes a more refined method for identifying the infective processes in prosthetic valves. This could lead to more accurate diagnoses, particularly in cases in which other typical signs of IE are not present.

Such a modification in diagnostic criteria could have far-reaching effects on patient care, potentially improving the accuracy of IE diagnoses and, consequently, the appropriateness of treatment strategies. This study represents a significant step forward in the field of cardiac pathology and infectious disease management.

**Limitations**: The retrospective design of this study had some limitations, particularly regarding data completeness. Some patient records may have been incomplete or had missing crucial information, potentially reducing the findings. These missing data could have affected various aspects of the study, from patient characteristics to outcome measures, potentially limiting the comprehensiveness of the analysis. 

The limited number of participants may not adequately represent the broader patient population, potentially leading to skewed results or overlooking important subgroup variations. Additionally, conducting the study at a single center raises concerns about the applicability of the findings to other healthcare settings that may have different patient demographics, treatment protocols, or resource availability. To address these limitations and validate the study’s conclusions, it is imperative to conduct larger multicenter studies that can provide a more comprehensive and representative dataset, ultimately strengthening the reliability and external validity of the findings.

## Data Availability

The data presented in this study are available on request.
